# Prevention of postpartum depression via a digital acceptance and commitment therapy-based intervention: protocol for a pilot usability study

**DOI:** 10.3389/fpsyt.2025.1633229

**Published:** 2025-11-28

**Authors:** Silvia Rizzi, Anna Elena Nicoletti, Chiara De Luca, Stefania Poggianella, Carlo Dalmonego, Claudia Paoli, Debora Marroni, Barbara Burlon, Vanda Chiodega, Martina Dallafior, Riccardo Pertile, Silvia Rizzi, Laura Valentini, Irene Alberi, Stefano Forti, Fabrizio Taddei

**Affiliations:** 1 Digital Health Research, Centre for Digital Health & Wellbeing, Fondazione Bruno Kessler, Trento, Italy; 2 Istituto Pavoniano Artigianelli, Trento, Italy; 3 Transmural Obstetric Gynecological Department, Healthcare Trust of the Autonomous Province of Trento (APSS), Trento, Italy; 4 Operating Unit of Psychology, Healthcare Trust of the Autonomous Province of Trento (APSS), Trento, Italy; 5 Department of Clinical and Evaluative Epidemiology, Healthcare Trust of the Autonomous Province of Trento (APSS), Trento, Italy

**Keywords:** digital health, perinatal mental health, postpartum depression, Acceptance and Commitment Therapy, virtual coach, User engagement

## Abstract

**Introduction:**

The perinatal period — encompassing pregnancy and the first months after childbirth — is a time of increased psychological vulnerability. It is often associated with high levels of anxiety, stress and depression. Access to psychological support is frequently limited by stigma, geographical barriers, and a shortage of services. Digital health interventions offer promising solutions to overcome these obstacles.

**Methods:**

This study evaluates the acceptability, feasibility, and user experience of REA, a virtual coach based on Acceptance and Commitment Therapy (ACT), to promote psychological well−being and prevent postpartum depression (PPD). Fifty pregnant women (25–30 weeks of gestation) will be recruited. The 8−week intervention delivers psychoeducational content via text, audio, and video, and collects steps, sleep, and heart rate via smartwatches for triangulation with self−reported measures. User Experience (UX) and User Engagement (UE) will be assessed with the System Usability Scale (SUS), the User Engagement Scale–Short Form (UES−SF), the Italian Chatbot Usability Scale, version B (ITA BUS B), and the User Version of the Mobile Application Rating Scale (uMARS), alongside semi−structured interviews. Psychological outcomes will be assessed pre–post with the two Whooley Questions, the Center for Epidemiological Studies Depression Scale (CES−D), the Multidimensional Psychological Flexibility Inventory (MPFI), and the 12−Item Short Form Health Survey (SF−12).

**Expected results:**

The intervention is expected to demonstrate high levels of user satisfaction and engagement (SUS ≥ 68, UES-SF ≥ 3,5/5; ITA BUS B ≥ 44/55 (≈4,0/5); uMARS ≥ 4,0/5), resulting in improvements in psychological flexibility, perceived well-being, and overall quality of life, recognizing that preventive efficacy will be evaluated in subsequent studies with controlled designs and postpartum outcome measures.

**Discussion:**

REA represents a scalable and accessible tool to support perinatal mental health, offering an innovative approach to the early prevention of postpartum distress.

**Trial registration:**

This study was approved by the ethics committee of the APSS (Provincial Health Services Authority) under number 12090 (May 15, 2025).

## 1 Introduction

### 1.1 Overview

The current body of scientific literature highlights an increasing need for psychological support among the general population, particularly among vulnerable groups exposed to high stress levels (1, 2). One of these vulnerable periods is the perinatal period, which is characterized by profound physical, psychological and social changes for women. How women adapt to these changes significantly impacts their quality of life and psychological well-being. Research indicates that pregnant women often experience psychological symptoms, with anxiety, stress, and depression being the most commonly reported (3, 4).

Estimates of postpartum depression (PPD) vary depending on symptom severity: postpartum blues (“baby blues”) is a transient and self−limiting mood disturbance in the early postpartum period that affects 30–80% of new mothers (5, 6). It is distinct from PPD, which affects about 10–15% of mothers (7) and is characterized by longer duration and/or greater severity with functional impairment. One challenge in treating PPD is that many mothers hide their symptoms, which is why it is often called “smiling depression” (8). Compared with other forms of depression, PPD includes distinctive symptoms such as confusion, auditory hallucinations, and delusions (9). Confusion is one of the most common and persistent symptoms (10), especially in severe cases (5, 11).

Although education and support have been proposed as preventive measures (5), few concrete programs have been implemented to date. Prevention is crucial because symptoms appear soon after childbirth and can significantly impact a child’s development, especially the mother-child bond in the first three months (9, 10, 12, 13).

While evidence supporting the effectiveness of psychological interventions for pregnant women is growing (14–16), significant barriers to accessing such care persist. Geographic limitations, the scarcity of trained professionals, and the stigma surrounding mental health issues often prevent women from seeking and receiving adequate support. For example, a recent Italian study on perinatal mental health practices revealed that 80% of Mental Health Departments (MHDs) lack structured care pathways, and Mother-Baby Units, which are essential for managing severe maternal mental health disorders requiring hospitalization, are unavailable in the country (17).

The World Health Organization (WHO) advocates for mental health promotion strategies that emphasize inclusivity and scalability. In this context, digital technologies have emerged as promising tools to address psychological well-being, as they offer accessibility, flexibility, and the potential to reduce barriers such as stigma and limited service availability (18).

This study aligns with the WHO’s strategic framework by evaluating the acceptability and feasibility of a digital intervention aimed at promoting psychological well-being and preventing perinatal mental health issues and PPD. Specifically, the intervention leverages third-wave cognitive-behavioral techniques, such as those based on Acceptance and Commitment Therapy (ACT), which have demonstrated their suitability for low-intensity, e-health-delivered interventions (19, 20). ACT is based on the idea that trying to change or avoid thoughts, feelings, or memories is often unhelpful (21). People tend to become “fused” with these private experiences, treating them as if they were facts. This can lead them to focus on changing what is actually out of their control, which distracts them from their true values and goals. ACT encourages the acceptance of these experiences, helping people reconnect with what matters to them. We selected ACT as the theoretical and intervention framework for four reasons. First, ACT directly targets psychological flexibility—a proximal mechanism in our preventive aims—and is measured in this study via the MPFI (19–21, 26). Second, ACT’s acceptance- and values−based strategies are well suited to addressing intrusive or distressing perinatal thoughts without pathologizing their mere occurrence (19–21). Third, ACT modules are amenable to low−intensity, self−guided, digitally delivered formats, consistent with our e−health design (19, 20). Finally, while various psychological interventions show promise in the perinatal period (14–16), ACT offers a coherent process−based approach aligned with prevention and scalable delivery. In addition, digital prevention programs have shown feasibility in perinatal populations, supporting our implementation choice (18).

In the case of PPD, the intense stress and changes after childbirth can lead to unexpected and disturbing thoughts or feelings. While some fusion with thoughts may not be harmful, fusion with unfamiliar or distressing experiences, such as “I wish I did not have this baby”, can have serious consequences. If the mother avoids or believes these thoughts, she may feel guilty or disconnected from her baby. Even if someone is not fully fused with all their thoughts, they might still strongly believe what they see or hear. This becomes problematic when those perceptions are false, such as in hallucinations. In PPD, hallucinations and delusions, especially auditory ones, are not uncommon (9). In this protocol, the term perinatal is employed to denote the entire continuum from pregnancy to the initial months following childbirth, a period that is characterized by vulnerability but also by opportunities for prevention. The intervention, which is administered during pregnancy, aims to enhance psychological flexibility, stress regulation and perceived well-being, thereby reducing the proximal risk factors for postpartum distress. Within this framework, the ACT-based digital intervention offers psychoeducational content and experiential practices to support perinatal adaptation and contribute to the prevention of postpartum distress and depression.

In summary, there is a need for scalable solutions that support perinatal mental wellbeing and reduce barriers to access. In consideration of the ACT theoretical rationale and the WHO framework on mental health promotion, the REA protocol is hereby presented. The REA protocol is a virtual coach integrated into TreC Mamma that delivers an 8-week psychoeducational program during the perinatal period. In the ensuing sections, we delineate the objectives, methodologies, metrics and analyses employed to evaluate its acceptability, feasibility, usability and engagement, in addition to pre-post psychological changes.

### 1.2 Theoretical model

The present study is positioned within the design and development cycle of the ORBIT model (Obesity-Related Behavioral Intervention Trials) (22). It involves the delivery of material based on a validated intervention in text, audio and video formats by a virtual coach (i.e. a digital assistant), identified as REA (the name “Rea” was chosen for the chatbot about the Greek goddess Rhea, daughter of Uranus and Gaia, who is venerated in mythology as the mother of the Olympian gods), implemented within the TreC Mamma application (app).

This intervention is positioned within the framework of behavior change interventions (23), which aim to enable women to acquire adaptive strategies for their psychological well-being.

In this context the term *intervention* is used to identify an application - the virtual coach - that periodically proposes, in a predefined (rule-based), structured and orderly manner, psychoeducational material in audio, text and video format, structured and reviewed by psychological professionals. The end user reads and manages this material autonomously; therefore, the virtual coach is used as a simple information transmission tool.

### 1.3 Goal of the study and research questions

The intervention is designed to enhance the improvement of women’s psychological well-being by helping them manage stressful situations more effectively through modules based on ACT principles and to prevent postpartum distress. The study also envisages collecting and using data from wearable devices (smartwatches, CE-marked and GDPR compliant) for triangulation with objective parameters (such as sleep quality or physical activity level) to support the women’s self-reported data.

The primary objectives of this proof-of-concept study are (1) to explore the User Experience (UX) and User Engagement (UE) of women interacting with the TreC Mamma application and the virtual coach, REA and (2) to assess, through semi structured interviews, participants’ experience with both REA and the application, as well as their emotional responses throughout the intervention.

The secondary objectives are to assess psychological well-being before and after the intervention, to administer self-assessment questionnaires at the beginning and end of the psychoeducational program, and to analyze the data collected via wearable devices. It is important to emphasize that the data collected from smartwatches on steps, sleep and heart rate are objective indicators of behavior and physiological status, and are used to triangulate self-reports. This data has exploratory and non-causal value.

## 2 Methods

### 2.1 Design and setting

REA, the virtual coach developed within the TreC Mamma application, will interact with women for 8 weeks starting from week 25/30 of pregnancy, with one month between the end of session 6 and the beginning of session 7.

Specifically, there will be one psychoeducational session per week with integrated exercises. The woman can choose the day and time that best suits her needs to interact with REA.

At the beginning of the course, REA will administer the 4 self-report questionnaires, namely the two Whooley’s Questions (24), the Centre for Epidemiological Studies-Depression Scale (CES-D) (25), the Multidimensional Psychological Flexibility Inventory (MPFI) (26) and the 12-Item Short Form Survey (SF-12) (27) to establish the baseline level.

After the data collection phase, the intervention will be administered, and its contents will be presented in different formats (text, images, audio, and video tracks).

The questionnaires will be further administered at the end of the intervention to assess whether the women perceived a change in psychological well-being and quality of life. These data will then be triangulated with the parameters collected by the smartwatch.

Four questionnaires will be administered in order to assess usability: User Experience (UX) and User Engagement (UE). Usability and engagement measures include the User Engagement Scale Short-form (UES-SF) (28), the System Usability Scale (SUS) (29), the Italian version of the Chatbot Usability Scale (ITA BUS B) (30), and the User Mobile Application Rating Scale (uMARS) (31). The questionnaires are managed by a microservice within the TreC platform that complies with the requirements and regulations in terms of personal data protection.

After two months, women who have given consent will be undergoing a semistructured interview in order to investigate their experience in using REA and the TreC Mamma application, and to understand how the women felt during the process.

### 2.2 Participants, eligibility criteria, and screening

The study’s target population includes all pregnant women between 25 and 30 weeks of gestation attending the Pregnancy Care Service of the APSS in Trentino. Midwives from the APSS hospital and territorial service were involved in the study to identify women potentially eligible to participate.

Inclusion criteria to participate in the study are as follows: (1) being pregnant, (2) being in a gestational state between the nineteenth and twenty-sixth week, (3) being ≥ 18 years of age, (4) having a smartphone with Internet access, being able to download the app and being able to use it, (5) being a resident of the Autonomous Province of Trento, (6) knowing and understanding the Italian language, and (7) negative Whooley questions.

The exclusion criteria are as follows (1) patients unable to provide informed consent (a prerequisite for participation in the study), (2) inadequate understanding of the Italian language, (3) possible depression, (4) suicidal tendencies, (5) already being in/being in a psychological/psychotherapeutic pathway at the time of recruitment, (6) substance addiction/being in recovery for less than one year, and (7) a clinical history of previous psychological distress.

**Figure 1:**
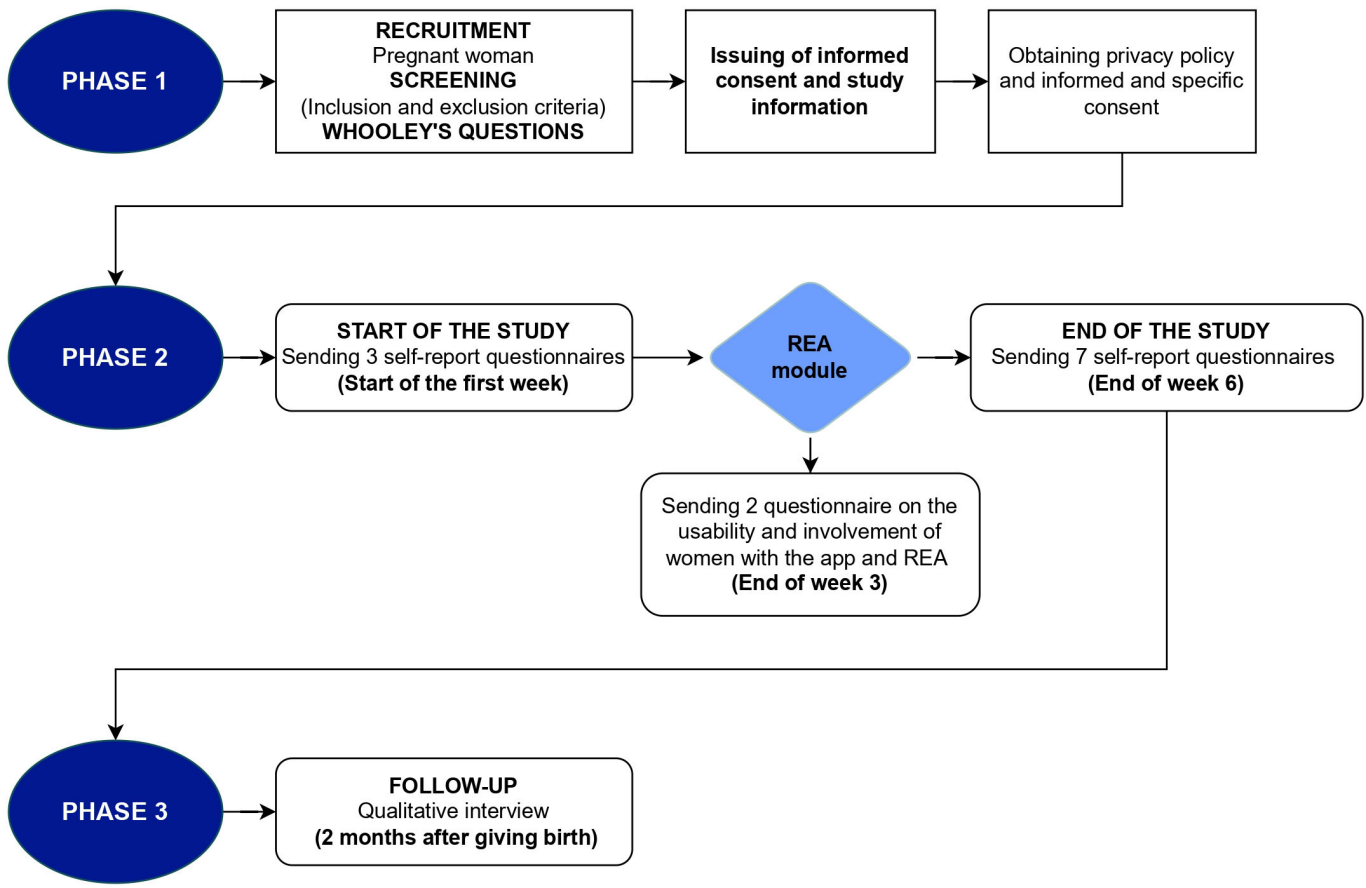
Flowchart of the research project procedure.

Recruited women who meet the inclusion criteria and give their consent to participate in the study will be enrolled in the study. All women who freely decide to take part in the study will be asked to sign an informed consent at the time of enrolment. After careful explanation of the study, its aims, how the data will be collected, managed and processed, the level of involvement required and the duration of the research, as well as any confidentiality issues. The woman will also be informed of the possibility of leaving the study at any time she wishes, without this in any way compromising the quality of care or interfering with her course of treatment, and without giving any explanation. The informed and conscious consent of the subject involved must be provided freely, voluntarily and in writing together with the data processing authorization form, prior to participation in the study. The women will be provided with the smartwatch and will sign the user agreement. The women will also receive an information sheet regarding the processing of personal data during the study. Copies of the informed consent and privacy policy regarding the study will be issued to the woman and will be available in the App. **Figure 1** shows a flowchart of the research project procedure.

### 2.3 REA intervention

The virtual coach, REA, delivers an intervention based on ACT techniques, also inspired by Espen Klausen’s manual (32). The intervention is designed to promote quality of life and perceived psychological well-being while also aiming to prevent psychological distress and PPD in pregnant women through a psychoeducational approach. The module is self-administered and available on both the Android and iOS platforms. All the contents of the dialogues have been developed by a group of psychologists belonging to the Digital Health Research unit of the Fondazione Bruno Kessler (FBK) in Trento and psychologists belonging to the Istituto Pavoniano Artigianelli in Trento, with specific competences in the field of communication, in collaboration with an ACT expert psychologist/psychotherapist, starting from the Handbook (ACT) for the prevention of distress and PPD (32). The content was subsequently reviewed by a senior psychologist from the Operative Psychology Unit of Trento’s Azienda Provinciale per i Servizi Sanitari (APSS).

**Figure 2:**
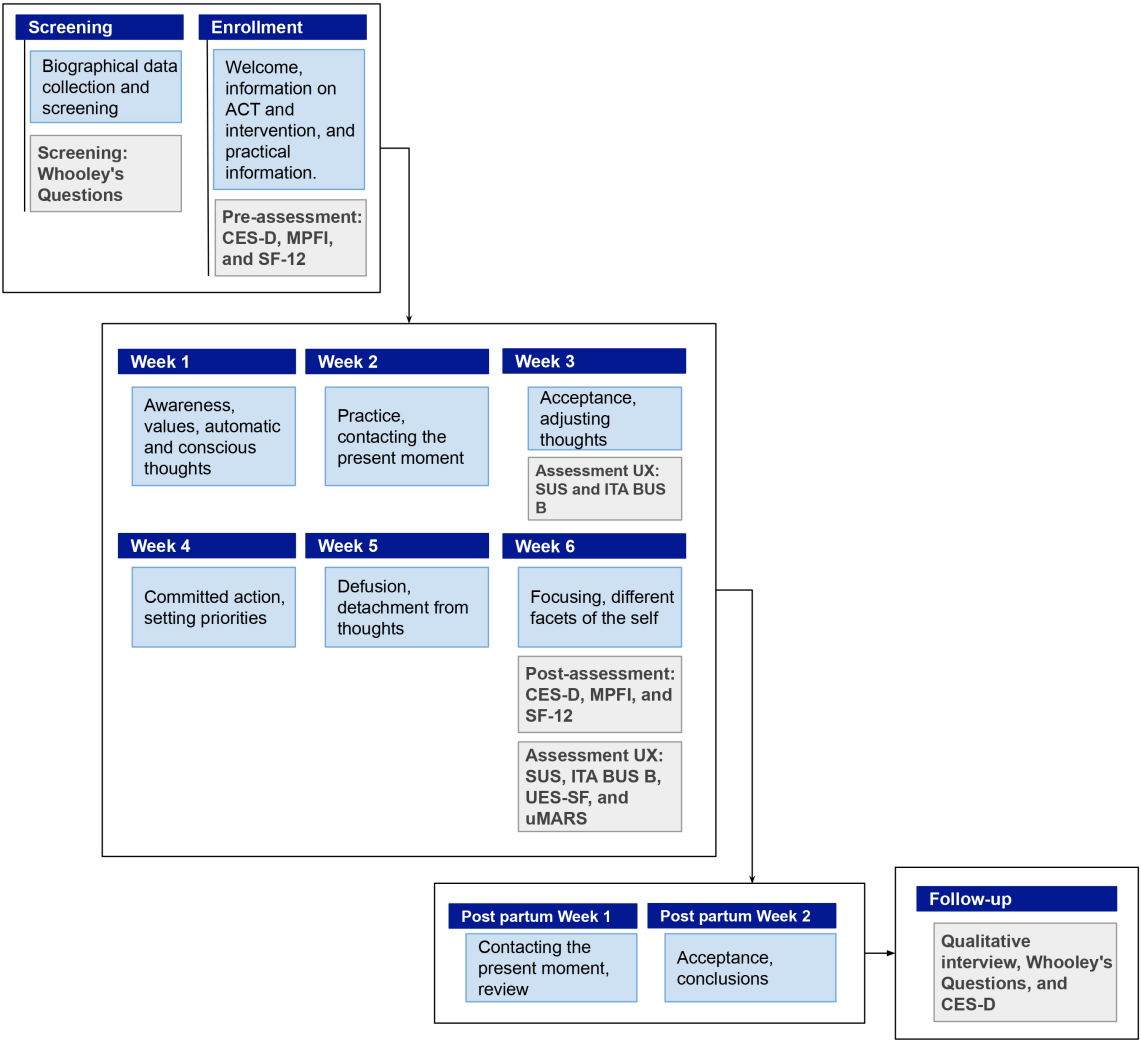
Graphical representation of the conversational protocol delivered to women and its chronological structure.

The intervention spans 8 weeks, with 6 weeks before and 2 weeks after delivery (see **Figure 2**). Each session lasts approximately 20 minutes.

The intervention aims to increase psychological flexibility in women through exercises based on the ACT model, thereby helping to prevent postpartum distress.

The content is delivered through a step-by-step process designed to promote self-awareness, acceptance and normalization of internal states, stress management and an overall improvement in psychological well-being.

The woman will also be provided with a smartwatch to be worn for the duration of the intervention, which collects the following parameters: steps, sleep and heart rate, which can be used for triangulation with the data self-reported by the woman in the questionnaires provided by the chatbot. The objective nature of the parameters collected by the smartwatch (steps, sleep, heart rate) is to serve as a supplementary measure to self-reports, with the aim of facilitating exploratory triangulation of behavioral patterns with psychological changes. It is imperative to note that these parameters will not be utilized for the purpose of making causal inferences or clinical decisions.

#### 2.3.1 Technological tools

The study’s technological component is based on the TreC platform, which allows citizens of the Autonomous Province of Trento to access, manage and share information on their health and well-being (33). TreC stands for ‘Cartella Clinica del Cittadino’ (citizen’s medical record) and is a reliable and well-formed platform designed to be a ‘system of systems’ rather than a mere data hub. TreC is designed with a flexible architecture, which enables the collection and management of heterogeneous data and allows the development and use of further subsystems to provide additional and specific functions.

For the present research project, an additional module of the TreC platform, TreC Mamma, is utilized. The application can be downloaded via a specific link sent directly to participants. Authentication takes place via a digital identity service (SPID or CIE).

**Figure 3:**
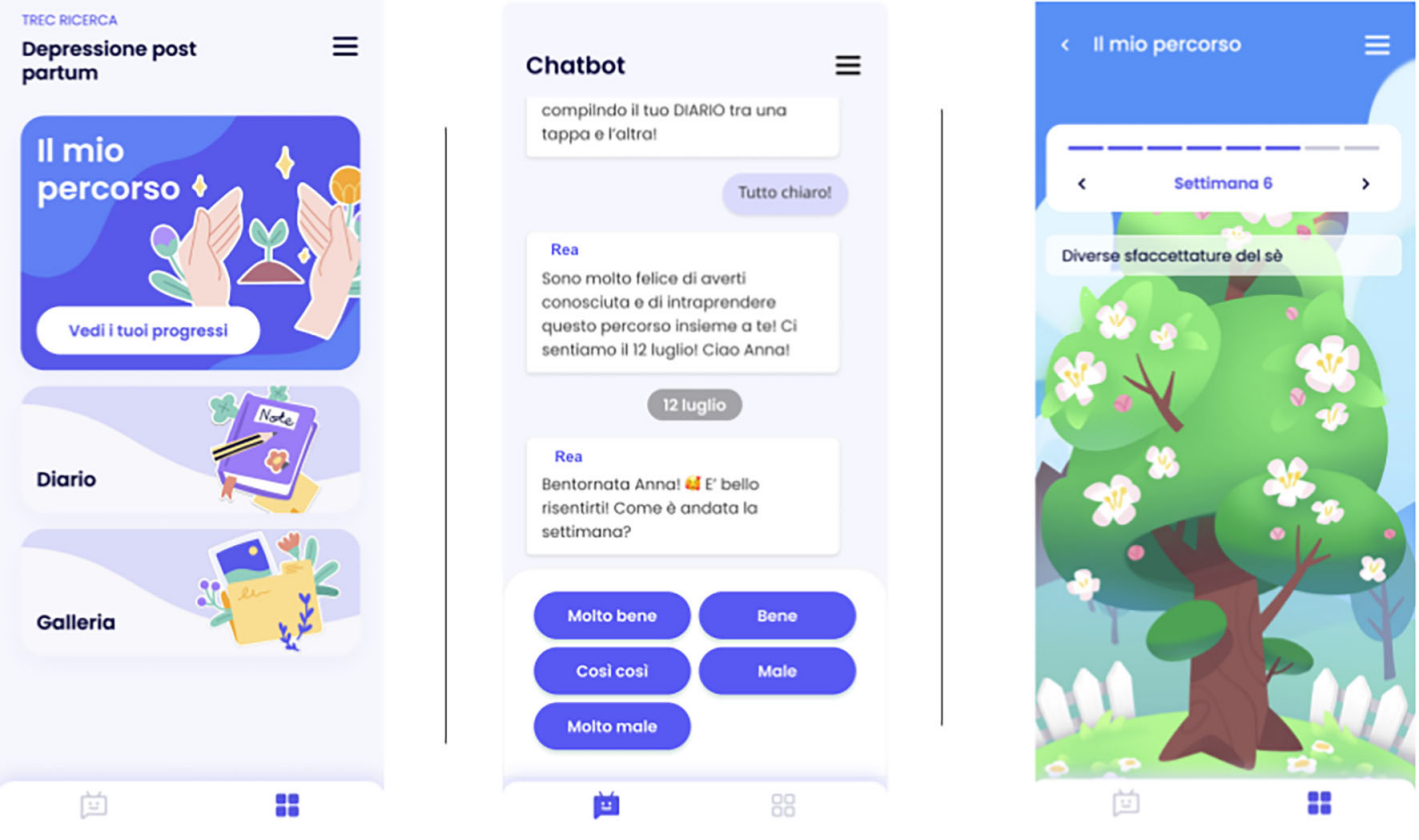
Mockups of the REA intervention application with some of its different sections.

The smartwatches used to conduct the study are CE-marked, nonmedical devices and GDPR compliant. **Figure 3** shows mockups of the REA intervention with some different sections.

### 2.4 Study outcome

#### 2.4.1 Primary outcomes

The primary outcomes are UX and UE. They will be assessed via questionnaires, namely the User Engagement Scale Short-form (UES-SF) (28), the System Usability Scale (SUS) (29), the Italian version of the Chatbot Usability Scale (ITA BUS B) (30), and the User Mobile Application Rating Scale (uMARS) (31), which will be administered at different time slots through the study, in particular the usability questionnaires will be administered at the end of the third (3 questionnaires) and sixth (end of the study; 4 questionnaires) weeks.

The endpoints to be measured are as followed:

- Scores of the individual items of the questionnaires;
- Mean score of the questionnaires;
- Differences between the average score values of the individual questionnaires at the different survey times.

The experience of using the app and the virtual coach (REA) and using the intervention itself will be assessed through a semi-structured interview. The endpoints to be measured are qualitative.

#### 2.4.2 Secondary outcomes and follow-up

The variable expressing the outcome ‘psychological well-being’ will be assessed by administering the 4 self-report questionnaires, namely the two Whooley’s Questions (24), the Centre for Epidemiological Studies-Depression Scale (25), the Multidimensional Psychological Flexibility Inventory (26) and the 12-Item Short Form Survey (27) at the beginning and end of the psychoeducational intervention (refer to **Table 1** for a detailed overview of the questionnaire adopted). The endpoints to be measured are as follows: a score of the individual items of the questionnaires, the average score of the questionnaires, and the difference between the average score values of the individual questionnaires at the different survey times. At the follow-up appointment, which takes place two months after childbirth, Whooley’s Questions will be administered. If the participant confirms her willingness, the CES-D will also be administered to assess the risk of PPD.

**Table 1:** Summary of the questionnaires administered and their timing.

Screening	At the beginning of the study (week 0)	At the end of week 3	At the end of the study (week 6)	Follow-up (2 months after birth)
Whooley’s Questions	–	–	–	Whooley’s Questions
–	CES-D^a^	–	CES-D	CES-D
–	MPFI^b^	–	MPFI	–
–	SF-12^c^	–	SF-12	–
–	–	SUS^d^	SUS*	–
–	–	ITA BUS B^e^	ITA BUS B*	–
–	–	–	UES-SF^f^*	–
–	–	–	uMARS^g^*	–
–	–	–	–	Qualitative interview
				

a CES-D, Center of Epidemiological Studies-Depression Scale.

b MPFI, Multidimensional Psychological Flexibility Inventory.

c SF-12, Short Form Health Survey 12.

d SUS, System Usability Scale.

e ITA BUS B, Chatbot Usability Scale, version B.

f UES-SF, User Engagement Scale-Short Form.

g uMARS, User Mobile Application Rating Scale.

*These usability questionnaires will be administered the following day, so as not to burden the participant during completion.

### 2.5 Data collection

The midwives and doctors involved at the time of enrolment will collect socio-demographic data, with the help of a pre-structured form, to which a unique alphanumeric code will be attributed. The socio-demographic data collection form and the data collected during the course of the study will be stored separately. The parameters requested from the women will be (1): date of birth (2), expected date of delivery, (3) educational qualification, (4) occupation, (5) marital status, (6) number of pregnancies and deliveries, and (7) partner’s occupation, if any.

The woman will also be asked if she would like to participate in a qualitative interview (see Appendix 2 for the interview structure) two months after the birth. If she agrees, she will be asked for a telephone number to be contacted again. Women who give consent to this interview will be sent (again via the app) a questionnaire one month after the expected date of birth in which the following will be asked: [1] actual date of birth, [2] sex of the child, [3] name of the child, and [4] date and time the woman wishes to be contacted for the interview. Finally, the woman will be offered the use of the smartwatch as an optional choice.

Overall, as shown in **Table 1**, the psychoeducational course involves the completion of self-report questionnaires, which are delivered at the beginning, during and at the end of the interaction with REA. The instrument’s completion time is approximately 15 minutes.

Specifically, 4 questionnaires will be administered at the beginning and at the end of the psychoeducational intervention to investigate depressive symptoms, psychological flexibility and quality of life. None of these questionnaires have a diagnostic purpose, so they will not be used to diagnose psychopathology but only to collect descriptive data. Women with psychopathology will be excluded *a priori* from participation in the study (as defined in the Exclusion Criteria section).

A further 4 questionnaires will be sent out during the course to analyze the usability and engagement of the woman with the app and the virtual coach, REA.

The Whooley questions (24) are based on two PHQ-9 (34) items with a yes/no answer mode, administered by the practitioner; a positive test is defined as a positive answer to even one question. The questions are: During the last month, have you often felt down, depressed or hopeless? Have you often felt little interest or pleasure in doing things in the last month?

In pregnancy, for the identification of ‘mixed’ depression the instrument has 100% sensitivity with 68% specificity. In the postnatal period, sensitivity is 100% with specificity 64% for ‘mixed’ type depression and 100% and 44% respectively for major depression.

The Centre for Epidemiological Studies-Depression Scale (CES-D) (25) is a 20-item measure that asks carers to rate how often in the last week they have experienced symptoms associated with depression, such as restless sleep, poor appetite and feelings of loneliness. Response options range from 0 to 3 for each item (0 = rarely or not at all, 1 = sometimes or a little, 2 = moderately or a lot, 3 = most of the time or almost always). Scores range from 0 to 60, with high scores indicating major depressive symptoms. Being an epidemiological instrument, it is particularly short; in fact, the administration takes about ten minutes.

The Multidimensional Psychological Flexibility Inventory (MPFI) (26) is an assessment tool that measures psychological flexibility on several dimensions. It investigates how people adapt their thoughts and behaviors in response to changing situations and challenges. The MPFI focuses on various aspects of psychological flexibility, including openness to experiences, present-moment awareness, and value-driven action. The 12 dimensions of flexibility and inflexibility (according to the Hexaflex model) are assessed through 60 Likert scale items from 1 to 6. The Italian adaptation of the MPFI scale was developed by Landi et al. (35).

The 12-Item Short Form Survey (SF-12) (27) consists of 12 items (taken from the 36 items of the original SF-36 questionnaire (36)) that produce two measures relating to two different aspects of health: physical health and mental health. The SF-12 consists of 4 scales (physical functioning, role and physical health, role and emotional state, mental health) each measured by 2 items and 4 scales each measured by 1 item (physical pain, vitality, social activities and general health). The subject is asked to respond about how he or she feels and how well he or she is able to perform usual activities, evaluating the day on which he or she completes the questionnaire and the previous 4 weeks.

The Italian Version of the Chatbot Usability Scale, version B (ITA BUS B) (28), is designed to assess users’ ease of use, effectiveness, and satisfaction in interacting with chatbots. This tool is structured around 11 items that cover various aspects of the user experience with chatbots, aiming to provide an in-depth, multidimensional assessment of their usability. The items involve responses on a 5-point Likert scale, where 1 equals “strongly disagree” and 5 equals “strongly agree” thus scoring from 11 to 55.

The System Usability Scale (29) is a quick and reliable tool for assessing system usability. It consists of 10 items with Likert-type responses from 1 to 5. The questionnaire, developed by Brooke, provides an overall score that reflects the ease of use and applicability of a system or product. This score can range from 10 to 50. A document with printable SUS questions is available online (37).

The User Engagement Scale Short-form (28) is a short self-report questionnaire to assess user engagement with a digital solution. This measure includes 12 items based on a 5-point Likert scale, ranging from 1 (strongly disagree) to 5 (strongly agree). The questionnaire consists of 4 factors: (1) focused attention, which indicates the feeling of being immersed in the interaction; (2) perceived usability, which is the negative effect experienced due to the interaction and the effort expended; (3) aesthetic attractiveness, which represents the graphical and visual appeal to a digital solution; (4) the reinforcement factor (reward). The latter is a single factor that includes duration, which evaluates the overall success of the interaction; novelty, which examines the general interest related to the interaction with a digital solution; and, finally, the perceived engagement factor, which evaluates the overall enjoyment of the interaction. This questionnaire was not translated into Italian and was, therefore, translated through the Back Translation procedure.

The User Mobile Application Rating Scale (31) is a tool for assessing the quality of mobile apps and its functionalities. It is characterized by a 20-item measure that includes 4 objective quality subscales (engagement, functionality, aesthetics, and information quality) and 1 subjective quality subscale rated on a 5-point Likert scale, ranging from 1 (poor) to 5 (excellent). The total and subscales scores have very high Cronbach alpha coefficients (.90 and.78-.80, respectively). The scale has been also validated in an Italian context (38).

Psychologists from the project team will conduct the interviews. The interviews are structured with *ad hoc* items to purposively capture specific characteristics of the study and participants’ experiences (39). This interview will be carried out two months after the birth, will last approximately 20 minutes and, with the woman’s consent, will be audio-recorded to allow subsequent analysis.

### 2.6 Sample size

The present research project is a proof-of-concept study, in which a limited sample is sufficient to achieve the intended aims.

If non-parametric statistics are conducted (assuming that the distribution is not normal), the sample should consist of N = 24 pregnant women with Bonferroni correction (α = .025).

The sample size and power for nonparametric tests (Kruskal-Wallis Test, Wilcoxon *post-hoc* test) were calculated according to Noether (40).

If parametric statistics are conducted (assuming that the distribution is normal), the sample must instead consist of 41 pregnant women with Bonferroni correction (α = .025).

Therefore, setting the power level at.80 and the significance level at.025 and taking into account a 20% possible drop-out, a total of 50 pregnant women were estimated to need to be recruited in order to carry out the study.

### 2.7 Data analysis

Preliminary data analysis is scheduled for early November 2025, while the final results will be available at the end of the study. The results will be published within one year after the conclusion of the study.

#### 2.7.1 Quantitative analysis

Statistical data processing will be conducted via R software, version 4.0.0 (RStudio Team, 2020), IBM SPSS Statistics (IBM Corp, 2020), Stata17 (StataCorp, 2021), and JASP (Jasp Team, 2024).

Categorical variables will be summarized through absolute and percentage frequency distributions, and quantitative variables through appropriate centrality and variability indices.

Descriptive analyses will be calculated for both the psychological variables (depressive symptoms, psychological flexibility and quality of life) and the usability (UX) and user involvement (UE) variables. These analyses will be performed on the variables analyzed at the beginning, during and at the end of the interaction with REA.

The relationships between variables will be analyzed mainly through *ad-hoc* statistical tests, such as the chi-square test, Fisher’s exact test, t-test for paired data or Wilcoxon’s nonparametric tests and the sign test (based on an assessment of compliance with assumptions) to understand the differences between the beginning and the end of the path in the same study sample to the variables under investigation.

In addition, univariate logistic or multinomial regression models will be presented. Finally, to eliminate possible confounding factors, multiple regression models will be proposed in which the effects of the explanatory variables on the outcome variables will be adjusted for possible confounders. For each analysis, statistical significance will be found with a p-value ≤.05.

Multivariate models will include adjustment for potential confounders collected in the protocol, including maternal age, educational attainment, employment status, marital status, previous pregnancies/births (parity), gestational age at enrolment, baseline CES-D, MPFI and SF-12 scores. The extant literature identifies these variables as being associated with perinatal mental well-being and adherence to digital solutions. Sensitivity analyses will explore the robustness of the results with respect to different model specifications and the availability of wearable data (steps, sleep, HR) as exploratory covariates.

#### 2.7.2 Qualitative analysis

About the analysis of the semi-structured interviews, a text mining approach (35) will be used to extract the answers that appear repeatedly from the interviews that will be conducted concerning the women’s experience of interacting with REA and with respect to how they felt throughout the process.

### 2.8 Ethical considerations and privacy

This study received approval from the Ethics Committee of the APSS (12090; May 15, 2025). At recruitment, all women who voluntarily choose to participate will be asked to sign an informed consent form after receiving a thorough explanation of the study, its objectives, the level of involvement needed, the duration of the research, and all ethical issues related to confidentiality. While the health professionals will provide part of the explanation, a video recorded by the research team will also be available, clearly detailing the study, its purpose, and the participants’ involvement in simple, understandable terms. The participants will be informed of the study’s results through the app.

Copies of the informed consent and privacy policy related to the study will be issued to the woman and will be available in the App.

Personal data is processed for research purposes. The functionalities of the platform and the virtual coach are intended to promote the psychological well-being of pregnant women.

Personal data are processed for research purposes within the scope of public interest tasks and against explicit consent for special categories of data, contact data and interview recordings.

All data collected will be kept confidential, and data necessary for the evaluation of the study objectives will be pseudo-anonymized before being processed.

The study manager undertakes to produce a report on the study and to ensure that the data is reported responsibly and consistently. Personal data will neither be disclosed nor disseminated, except in anonymized or aggregated form. The publication of data resulting from this study will take place regardless of the results obtained. The transmission or dissemination of the data, by means of scientific publications and/or presentations at congresses, conferences and seminars, will take place exclusively in anonymous form using only aggregated data that prevent the identities of the participants from being traced, even indirectly. This sharing is expected within one year after the conclusion of the study.

## 3 Expected results

The psychoeducational pathway is expected to yield significant results, primarily in terms of women’s satisfaction and engagement with the use of the innovative digital solutions (SUS ≥ 68; average UES-SF ≥ 3.5/5; ITA BUS B ≥ 44/55; uMARS ≥ 4/5). Thresholds for usability and engagement were pre−specified based on established interpretive benchmarks and validated scale ranges: SUS ≥68 reflects above−average usability; UES−SF ≥3.5/5 indicates engagement above the neutral midpoint; ITA BUS B ≥44/55 corresponds to an average item agreement of ≥4/5; and uMARS ≥4/5 denotes high app quality. These targets are consistent with guidance and validation for the respective instruments (28–31).

We anticipate an increase between pre- and post-intervention in the levels of psychological well-being, quality of life of the woman and psychological flexibility. Specifically, we hypothesize: (i) an increase in MPFI psychological flexibility, (ii) an improvement in the mental component of the SF-12, (iii) a reduction in CES-D. These outcomes are exploratory and consistent with the main objective of assessing the acceptability, feasibility, and UX/UE of the intervention. It is important to highlight that, on the one hand, REA can be a valuable resource in ensuring regular psychoeducational support for women during the perinatal period. On the other hand, the literature has shown that women during the perinatal period indicated a preference for support provided online, suggesting that the implementation of digital interventions can overcome barriers around social stigma and reluctance to seek help.

### 3.1 Risks and mitigations

Possible critical issues that may arise during the study include: (i) low adherence to the program (mitigation: in-app reminders and flexibility of use); (ii) digital overload (mitigation: short sessions ≈20 minutes, alternating formats, burden monitoring); (iii) emergence or worsening of symptoms during the intervention (mitigation: upstream exclusion via Whooley, clear messages on help channels, clinical escalation where necessary); (iv) selection/adherence bias (mitigation: detailed description of the sample and sensitivity analysis); (v) limitations of wearable data (mitigation: use for exploratory triangulation, not causal inferences).

## 4 Discussion

This study aims to investigate and evaluate women’s experience and engagement with the TreC Mamma App and the virtual coach REA and assess the level of pre-post psychological well-being.

Firstly, we expect to collect relevant feedback and suggestions from the women to improve the structure of the intervention, making it more engaging and improving the interaction. Furthermore, we contemplate finding differences in terms of improved pre-post psychological well-being. However, we know that this change may not be related to the intervention itself and that the effectiveness evaluation will be carried out through a randomized clinical trial later.

All results will be reported and adequately discussed, including through a comparison with the relevant literature.

In the theoretical model that has been adopted, the promotion of well-being and psychological flexibility is considered to be a proximal goal of ACT approach (19–21); the prevention of PPD is regarded as a distal goal (24), which is mediated by these mechanisms. The present pilot phase is centered on the assessment of acceptability and usability, in addition to the collection of preliminary pre-post signals. It is acknowledged that for a more comprehensive evaluation, an RCT incorporating structured postpartum measures is required.

### 4.1 Limitations

This study has several limitations that should be acknowledged and addressed in the implementation of future studies. First, as mentioned above, it is crucial to consider that this study does not aim to evaluate the effectiveness of the intervention, which is why a control group is not planned. It will be crucial to involve a control group to assess the actual effectiveness in an RCT study. Moreover, the present study consists of several pregnant women from the Autonomous Province of Trento, in Italy. Expanding the study to include participants from other Italian regions—and potentially other countries—would enhance the generalizability of the findings.

It is important to highlight that REA might be a valuable technological support in providing regular psycho-educational services for women during the perinatal period. Previous research has indicated that women during the perinatal period preferred online support, suggesting that implementing digital interventions can overcome barriers to social stigma and asking for help. Finally, it is essential to emphasize that this technological support (REA and TreC Mamma) is not a substitute for clinical and medical pathways and in-life medical support; instead, it is an additional element in promoting psychological well-being during the perinatal period.

The absence of a control group limits causal inference; a subsequent randomized trial is therefore needed in line with the ORBIT model for behavioral intervention development (22). Moreover, generalizability should be examined beyond the study’s single−region sample, considering variability in perinatal mental health pathways (17). Finally, while digital interventions may mitigate access barriers, acceptability and engagement can vary across contexts (18), underscoring the importance of future replication studies.

### 4.2 Conclusion

The current literature underscores a growing preference for online support among women in the perinatal period, highlighting the potential of digital interventions to address barriers related to social stigma and seeking assistance. In this context, REA emerges as a promising resource, offering consistent psychoeducational support for women throughout the perinatal period.
